# The IoT and AI in Agriculture: The Time Is Now—A Systematic Review of Smart Sensing Technologies

**DOI:** 10.3390/s25123583

**Published:** 2025-06-06

**Authors:** Tymoteusz Miller, Grzegorz Mikiciuk, Irmina Durlik, Małgorzata Mikiciuk, Adrianna Łobodzińska, Marek Śnieg

**Affiliations:** 1 Institute of Marine and Environmental Sciences, University of Szczecin, Wąska 13, 71-415 Szczecin, Poland; 2 Faculty of Data Science and Information, INTI International University, Nilai 71800, Malaysia; 3 Department of Horticulture, Faculty of Environmental Management and Agriculture, West Pomeranian University of Technology in Szczecin, Słowackiego 17, 71-434 Szczecin, Poland; grzegorz.mikiciuk@zut.edu.pl; 4 Faculty of Navigation, Maritime University of Szczecin, Waly Chrobrego 1-2, 70-500 Szczecin, Poland; i.durlik@pm.szczecin.pl; 5 Department of Bioengineering, Faculty of Environmental Management and Agriculture, West Pomeranian University of Technology in Szczecin, Słowackiego 17, 71-434 Szczecin, Poland; malgorzata.mikiciuk@zut.edu.pl; 6 Institute of Biology, University of Szczecin, 71-415 Szczecin, Poland; adrianna.lobodzinska@phd.usz.edu.pl; 7 Doctoral School, University of Szczecin, 71-412 Szczecin, Poland; 8 Department of Agroengineering, Faculty of Environmental Management and Agriculture, West Pomeranian University of Technology in Szczecin, Słowackiego 17, 71-434 Szczecin, Poland; marek.snieg@zut.edu.pl

**Keywords:** artificial intelligence, edge computing, internet of things, precision agriculture, remote sensing, sensor networks, smart farming, soil monitoring, sustainable agriculture, UAV-based imaging

## Abstract

The integration of the Internet of Things (IoT) and artificial intelligence (AI) has reshaped modern agriculture by enabling precision farming, real-time monitoring, and data-driven decision-making. This systematic review, conducted in accordance with the PRISMA methodology, provides a comprehensive overview of recent advancements in smart sensing technologies for arable crops and grasslands. We analyzed the peer-reviewed literature published between 2020 and 2024, focusing on the adoption of IoT-based sensor networks and AI-driven analytics across various agricultural applications. The findings reveal a significant increase in research output, particularly in the use of optical, acoustic, electromagnetic, and soil sensors, alongside machine learning models such as SVMs, CNNs, and random forests for optimizing irrigation, fertilization, and pest management strategies. However, this review also identifies critical challenges, including high infrastructure costs, limited interoperability, connectivity constraints in rural areas, and ethical concerns regarding transparency and data privacy. To address these barriers, recent innovations have emphasized the potential of Edge AI for local inference, blockchain systems for decentralized data governance, and autonomous platforms for field-level automation. Moreover, policy interventions are needed to ensure fair data ownership, cybersecurity, and equitable access to smart farming tools, especially in developing regions. This review is the first to systematically examine AI-integrated sensing technologies with an exclusive focus on arable crops and grasslands, offering an in-depth synthesis of both technological progress and real-world implementation gaps.

## 1. Introduction

The global agricultural sector is at a critical juncture, facing increasing challenges related to climate change, resource scarcity, labor shortages, and the need for higher productivity to sustain a growing global population [1,2]. Traditional agricultural methods, reliant on manual labor and experience-based decision-making, often result in inefficient resource use, unpredictable yields, and vulnerability to extreme weather events [3,4]. The integration of Internet of Things (IoT) technologies and artificial intelligence (AI) in agriculture offers an unprecedented opportunity to optimize farming operations, improve resource efficiency, and enhance resilience against environmental stressors [5,6].

The IoT, in the context of agriculture, refers to networks of interconnected sensors, actuators, and cloud-based platforms that enable the real-time monitoring and automation of critical agricultural processes [7,8]. These systems collect large-scale, high-resolution data on soil properties, climate conditions, crop growth, and pest infestations, facilitating data-driven decision-making [9,10,11]. AI further enhances these capabilities by employing machine learning (ML) and deep learning (DL) algorithms to analyze sensor data, predict future outcomes, and automate responses, reducing reliance on human intervention [12,13]. The synergy of IoT and AI-driven solutions enables precision agriculture, allowing farmers to apply water, fertilizers, and pesticides with optimal efficiency, thus reducing costs and minimizing environmental impact [14,15,16,17].

The importance of these technologies is underscored by their potential to increase agricultural productivity while reducing resource consumption, ensuring food security and sustainability [18,19]. Governments, research institutions, and agribusinesses worldwide are investing heavily in AI-powered analytics, IoT-enabled monitoring systems, and robotics to modernize farming practices [20]. However, despite their transformative potential, these technologies face significant challenges in implementation, particularly in large-scale arable crops and extensive grassland management, where environmental variables are more complex and difficult to control [15,21].

Smart sensing technologies have revolutionized agricultural monitoring and decision-making by providing real-time, high-precision data on key environmental and biological factors [22,23]. These technologies include a wide array of sensor systems, unmanned aerial vehicles (UAVs), satellite-based remote sensing platforms, and AI-driven predictive models. They enable farmers to monitor and respond to fluctuations in soil moisture, temperature, nutrient levels, and pest presence, reducing reliance on generalized farming practices that may not be suitable for specific local conditions [24,25,26].

In arable crop management, IoT-enabled sensors play a crucial role in precision irrigation, ensuring that crops receive the optimal amount of water while minimizing waste [27,28]. Soil sensors measure parameters such as pH, salinity, and nitrogen content, providing critical data for site-specific fertilization [29,30,31]. Machine vision and AI-powered disease detection systems allow the early identification of plant stress, enabling targeted interventions before significant yield losses occur [32,33,34].

Grassland management, both in rural and urban settings, also benefits from these technological advancements [35]. Smart sensing systems assist in monitoring vegetation [27,36,37] health, biomass estimation, and grazing patterns, facilitating sustainable pasture management. In urban and peri-urban environments, smart grassland monitoring contributes to landscape maintenance, ecosystem services, and biodiversity conservation [38,39,40]. Unlike controlled greenhouse environments, where conditions can be artificially regulated, arable crops and grasslands are subject to dynamic and unpredictable environmental factors, making real-time data collection and adaptive AI models essential for effective management [41,42,43,44].

While sensor-based systems have seen widespread adoption in controlled agricultural environments, their implementation in open-field farming and grassland ecosystems presents unique technical and economic challenges [39,45,46]. Wireless sensor networks (WSNs), IoT gateways, and cloud-based analytics platforms must be designed to operate efficiently in remote areas with limited connectivity, ensuring reliable data transmission and processing capabilities [47,48,49,50].

Traditional agriculture has long relied on historical knowledge, intuition, and manual labor for decision-making. Farmers often use fixed irrigation schedules, generalized fertilization plans, and broad-spectrum pesticide applications, which may lead to the overuse of resources, increased production costs, and environmental degradation [51,52,53,54]. In contrast, smart agriculture leverages real-time data analytics and AI-driven recommendations to optimize these practices, enabling site-specific and adaptive management strategies [17,55,56].

One of the biggest challenges in transitioning from traditional to smart agriculture is technological accessibility and cost [57,58]. While large-scale commercial farms have increasingly adopted IoT-based precision agriculture, smallholder farmers often struggle with the high cost of sensors, data storage, and AI-powered analytics platforms [59,60]. Additionally, infrastructure limitations in rural areas, including weak network connectivity and insufficient digital literacy, create barriers to widespread adoption [61].

Another critical challenge is data heterogeneity and interoperability. The agricultural sector generates massive volumes of data from diverse sources, including IoT sensors, satellite imagery, UAVs, and farm management software [62,63]. However, integrating these datasets into a unified decision-making framework remains complex, requiring standardized data formats, interoperability protocols, and AI-driven data fusion techniques [64].

Beyond technological and economic barriers, environmental and ethical concerns also need to be addressed [65]. The increased use of automated agricultural machinery, AI-driven decision-making, and autonomous systems raises concerns about the displacement of traditional farming jobs, data ownership rights, and algorithmic transparency [66]. Moreover, the energy consumption of IoT devices and electronic waste from sensor networks present additional sustainability challenges that must be managed [67,68].

### Research Objectives and Scope of the Review

Given the growing significance of the IoT and AI in modern agriculture, this systematic review aims to analyze and synthesize current research on smart sensing technologies for arable crops and grasslands. By employing a PRISMA-based methodology, this study systematically examines recent advancements, emerging trends, and key challenges in the field. This review is structured to answer critical research questions, including the following:

1. What are the most widely used smart sensing technologies in arable crops and grassland management?
2. How do AI and machine learning models enhance decision-making in precision agriculture?
3. What are the technical, economic, and environmental barriers to the large-scale adoption of IoT-based solutions?
4. What future research directions and innovations are needed to further improve smart agriculture?

The scope of this review is interdisciplinary, integrating knowledge from agriculture, environmental science, data analytics, and engineering. It includes an assessment of IoT infrastructure, AI-powered decision-making frameworks, sensor technologies, and case studies from real-world applications. The findings of this study aim to provide valuable insights for researchers, policymakers, and agribusiness stakeholders seeking to develop and implement scalable, sustainable, and cost-effective smart agricultural solutions.

By addressing both technological opportunities and existing barriers, this review contributes to the growing body of knowledge on data-driven agriculture, offering a comprehensive and evidence-based perspective on the future of IoT and AI in smart farming.

This review contributes to the literature by providing the first PRISMA-based synthesis focused specifically on the integration of AI and the IoT in arable crop and grassland systems. It identifies key technological trends, evaluates real-world deployments, highlights limitations in current approaches, and proposes research and policy recommendations to guide future development of sustainable smart agriculture.

## 2. Materials and Methods

### 2.1. Systematic Review Protocol

This study follows the Preferred Reporting Items for Systematic Reviews and Meta-Analyses (PRISMA) guidelines, ensuring a rigorous and transparent methodology for identifying, selecting, and synthesizing the relevant literature [69,70]. The PRISMA approach structures the review process into four distinct phases: identification, screening, eligibility, and inclusion [71,72]. This ensures that only high-quality, relevant studies are considered, minimizing bias and increasing reproducibility. The objective is to provide a comprehensive overview of the IoT and AI applications in agriculture, focusing on smart sensing technologies for arable crops and grassland management [70].

This systematic review was designed to answer critical research questions concerning the technological advancements, implementation challenges, and future potential of IoT and AI-driven smart agriculture systems [73,74]. By analyzing recent studies, this review aims to identify trends, highlight key areas of application, and assess the effectiveness of these technologies in precision irrigation, soil monitoring, disease detection, and overall crop and grassland management.

### 2.2. Research Questions

This review seeks to address several key research questions. Firstly, it aims to determine the most widely used smart sensing technologies in arable crops and grasslands. Understanding the dominant types of sensors and IoT architectures will provide insight into the current state of technological integration in agricultural settings. Secondly, it explores how artificial intelligence enhances decision-making in smart farming by examining the types of machine learning models and predictive algorithms utilized in agriculture-specific IoT ecosystems.

Another major focus is identifying the technical, economic, and environmental barriers to the large-scale adoption of IoT-based solutions. These barriers include connectivity limitations, data privacy concerns, high infrastructure costs, and challenges related to sensor calibration and integration. Lastly, this review investigates emerging trends and potential innovations that could shape the future of AI and IoT in agriculture, including edge computing, blockchain integration, and autonomous agricultural systems.

### 2.3. Scope and Eligibility Criteria

The scope of this review is interdisciplinary, incorporating research from computer science, environmental engineering, agronomy, and data science. It focuses on studies discussing IoT-enabled sensing technologies and AI-based analytical frameworks applied to arable crops and grassland ecosystems. While many smart agricultural technologies have been developed for greenhouses and permanent crops, this review specifically examines their applications in open-field settings, where environmental variability presents unique challenges.

The eligibility criteria were established to ensure the inclusion of high-quality, peer-reviewed research. Studies selected for analysis must focus on the IoT, AI, or sensor-based systems applied to agriculture. Research papers presenting real-world case studies, experimental validations, or significant theoretical contributions were prioritized. Papers that do not explicitly discuss sensor integration, AI-driven decision-making, or their impact on precision farming were excluded. Similarly, the non-peer-reviewed literature, such as patents, white papers, and policy reports, was omitted unless it contributed significant empirical evidence.

### 2.4. Search Strategy

To ensure a comprehensive dataset, a structured search was conducted across multiple scientific databases, including Scopus, Web of Science (WoS), IEEE Xplore, and SpringerLink. Additionally, Google Scholar was consulted for the gray literature to mitigate publication bias and capture recently published preprints. The search strategy involved a combination of controlled vocabulary and Boolean operators, optimizing query precision to retrieve relevant studies while minimizing irrelevant results.

A structured keyword search was performed using terms related to the IoT, AI, and smart agriculture. The Boolean search query applied was as follows:

*(“Internet of Things” OR iot OR “wireless sensor networks” OR wsn OR “smart sensors”) AND (“artificial intelligence” OR ai OR “machine learning” OR ml OR “deep learning” OR dl) AND (“agriculture” OR “smart farming” OR “precision agriculture” OR “arable crops” OR “grasslands” OR “meadow management” OR “urban grasslands”) AND (“sensor-based systems” OR “proximal sensing” OR “remote sensing” OR “optical sensors” OR “acoustic sensors” OR “electromagnetic sensors” OR “soil sensors” OR “water sensors” OR “climate monitoring” OR “crop monitoring” OR “precision irrigation” OR “precision fertilization” OR “pest management”)*

This query was executed on 20 March 2025, ensuring temporal relevance. The search results were filtered based on relevance, citation count, and recency, ensuring that only high-impact and thematically aligned research was included.

### 2.5. Study Selection Process

**Figure 1:**
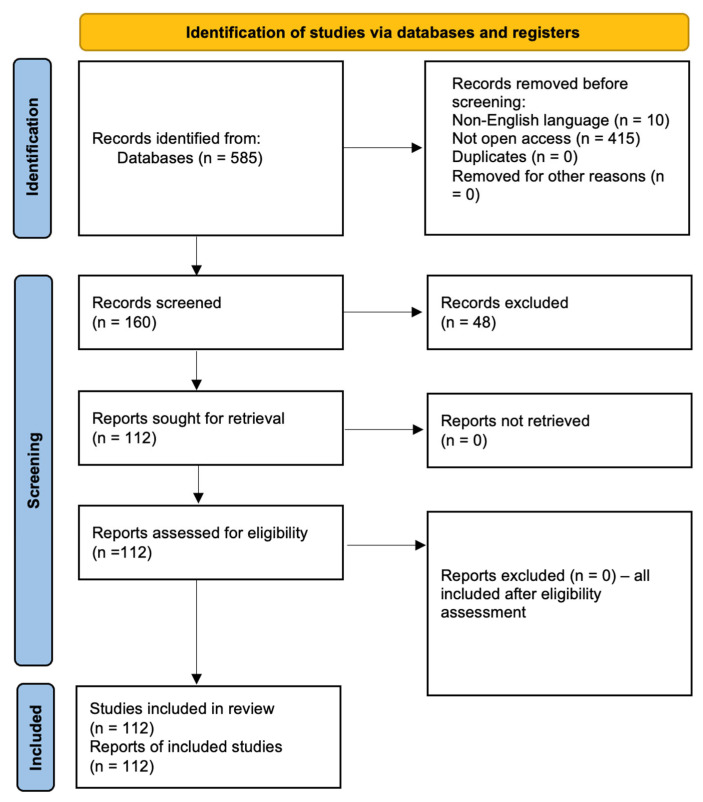
PRISMA flow diagram.

The study selection process followed a structured multi-stage approach. An initial search across major scientific databases including Scopus, Web of Science, IEEE Xplore, SpringerLink, and Google Scholar yielded 585 records. After excluding 10 non-English language publications, an open-access filter was applied, reducing the dataset to 160 publications. Subsequently, only peer-reviewed journal articles and conference papers were retained, which led to the exclusion of 48 non-research or gray literature documents. The final dataset consisted of 112 publications, which were used for qualitative and thematic analysis in this systematic review. A detailed breakdown of the screening process is presented in Table 1 and visualized using a PRISMA flow diagram (Figure 1).

### 2.6. Data Extraction and Categorization

Each selected study underwent a structured data extraction process, categorizing key technological, methodological, and agricultural attributes. The extracted parameters included the type of IoT architecture, sensor specifications, AI models used, agricultural application area, and performance metrics. Studies were classified into major categories, such as precision irrigation, disease detection, soil quality assessment, and grassland monitoring.

Another critical aspect of data extraction involved assessing the scalability, cost-effectiveness, and sustainability of the proposed solutions. Many IoT-based agricultural systems rely on high-cost proprietary hardware, making them less accessible to smallholder farmers. This review categorized studies based on whether they utilized open-source IoT platforms, low-cost sensors, or cloud-based AI models, as these factors influence real-world adoption rates.

### 2.7. Risk of Bias Assessment

Bias can significantly affect the reliability of systematic reviews. This study systematically assessed potential sources of selection bias, publication bias, technology bias, and data bias to ensure the validity of the findings.

Selection bias arises when certain types of studies are disproportionately included or excluded. Since this review focused on English-language publications from specific databases, there is a risk that important regional studies in other languages were overlooked. While efforts were made to include diverse sources, the exclusion of non-English papers remains a limitation.

Publication bias was also considered, as studies with positive results are more likely to be published than those with negative or neutral findings. To mitigate this, the gray literature was included where possible, ensuring that unpublished studies or preprints with valuable insights were not ignored.

Technology bias occurs when research disproportionately focuses on commercially available AI and IoT solutions, often funded by private agribusinesses. Studies promoting proprietary sensor technologies may emphasize performance benefits while overlooking limitations related to cost, compatibility, or scalability. This review critically examined funding disclosures and methodology sections to assess the objectivity of technological claims.

Data bias was another major concern, as many studies rely on region-specific datasets, making generalizability difficult. Smart agricultural systems developed in high-tech environments may not translate well to low-resource farming settings. The variability in sensor calibration, data acquisition methods, and machine learning training sets was carefully analyzed to determine the applicability of research findings across different agricultural contexts.

The comprehensive risk of bias evaluation served to strengthen the credibility of the review and guided the interpretation of the findings. The explicit consideration of bias types improved transparency and helped identify limitations in data generalizability, study funding, and regional scope.

### 2.8. Limitations and Challenges of the Review Process

Despite following a rigorous PRISMA methodology, several limitations remain. The heterogeneity of studies posed a challenge, as IoT and AI applications vary widely based on geographic, climatic, and technological factors. The differences in sensor specifications, AI model architectures, and validation methods made direct comparisons between studies difficult.

Another challenge was the evolving nature of the field. IoT and AI technologies are rapidly advancing, meaning that new innovations and breakthroughs may emerge that are not covered within the timeframe of this review. Additionally, since many highly specialized AI models in agriculture are developed by private companies, access to proprietary algorithms and datasets was limited.

Despite these challenges, this review provides a comprehensive, unbiased synthesis of the existing literature, offering insights into the current state, challenges, and future potential of the IoT and AI in arable crop and grassland management.

## 3. Results

### 3.1. Growth Trends in Research Publications

**Figure 2:**
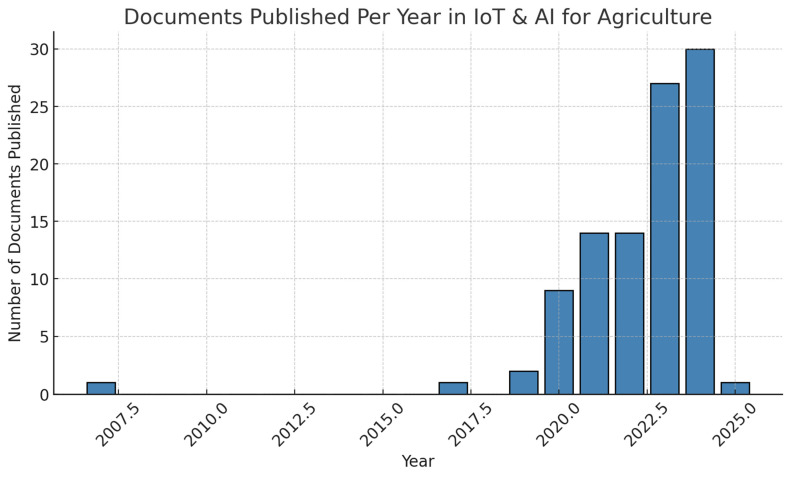
Documents published in study topic.

An analysis of the publication trends in IoT and AI applications in agriculture indicates a dramatic increase in research activity over the past five years. The distribution of peer-reviewed articles and conference papers by year reveals that the field has gained substantial momentum, particularly in the years 2022–2024 (Figure 2).

The results clearly indicate that before 2019, research in this domain was limited, with only isolated studies exploring IoT-based applications in agriculture. However, from 2020 onwards, there was a rapid increase in publications, reflecting the growing interest in AI-driven smart farming solutions. The sharp rise in 2023 and 2024 suggests a growing acceptance of these technologies, driven by advancements in low-power IoT devices, cloud computing, and AI-based analytics.

### 3.2. Geographical Distribution of Research

**Figure 3:**
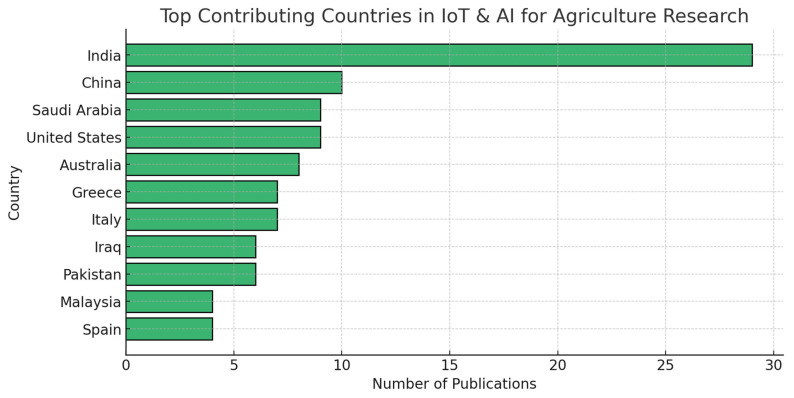
Top countries involved in studies of IoT and AI for agricultural research.

The global distribution of research contributions highlights significant regional disparities in IoT and AI adoption in agriculture. A review of publication data by country or territory reveals that India leads in research output, followed by China, Saudi Arabia, and the United States (Figure 3).

The concentration of research in India, China, and Saudi Arabia suggests that these countries have actively invested in smart agriculture initiatives, likely due to pressing agricultural challenges, government-funded research programs, and rapid digitalization efforts.

Interestingly, developed nations such as the United States, Australia, and the United Kingdom have also made significant contributions, reflecting a parallel trend of agritech adoption in both developed and developing regions. Meanwhile, European nations, including Greece, Italy, and Spain, have focused research efforts on integrating AI with sustainable agriculture and resource-efficient farming techniques.

The low research output from African and Latin American nations suggests that financial and infrastructural limitations may hinder IoT adoption in these regions, despite their potential to benefit significantly from AI-driven agricultural innovations.

### 3.3. Classification of Published Research

**Figure 4:**
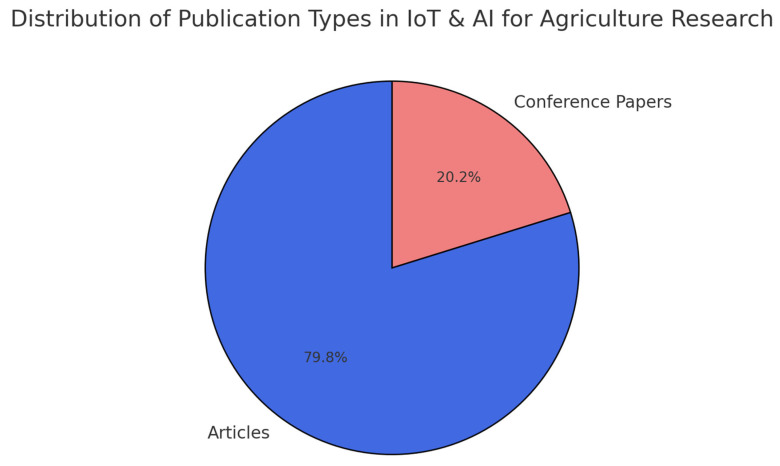
Distribution of paper type studied in article.

A detailed breakdown of the types of research documents published in this field indicates that journal articles constitute the majority of publications, followed by conference papers (Figure 4).

The dominance of peer-reviewed journal articles suggests a focus on empirical research, real-world applications, and the experimental validation of IoT-based agricultural systems. The presence of conference papers indicates ongoing theoretical discussions, emerging trends, and preliminary findings, particularly in computer science and engineering conferences.

### 3.4. Exclusions and Refinements in Dataset

During the literature selection process, certain exclusions were applied to refine the dataset and ensure high-quality analysis. Studies in languages other than English were excluded, resulting in the removal of 22 documents. Additionally, only peer-reviewed journal articles and conference papers were considered, leading to the exclusion of 112 non-research documents.

Furthermore, open-access filtering removed 268 publications, focusing the dataset on scientifically validated and rigorously reviewed studies. While the open-access literature often provides valuable insights, the decision to exclude it was based on concerns regarding variable peer-review standards and potential data reliability issues.

### 3.5. Citation Trends over Time

**Figure 5:**
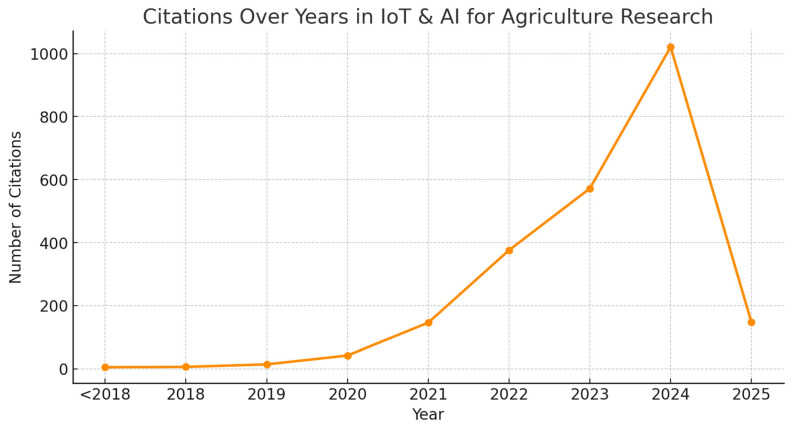
Citations of research involving IoT and AI usage in agriculture.

An examination of citation trends reveals that research in this domain has experienced progressive growth in academic impact. Early studies (before 2015) received minimal citations as the field had yet to gain prominence. However, from 2020 onwards, citation numbers increased exponentially, reflecting the growing recognition and integration of AI and the IoT in agricultural research (Figure 5).

The dataset analysis suggests that high-impact publications from 2022 to 2024 are driving significant advancements, particularly in precision irrigation models, AI-based crop disease prediction, and cloud-integrated IoT farming networks. The increasing number of citations indicates strong academic interest and suggests that the IoT and AI-driven smart agriculture is an evolving and rapidly expanding field.

### 3.6. Overview of Smart Sensing Technologies

**Figure 6:**
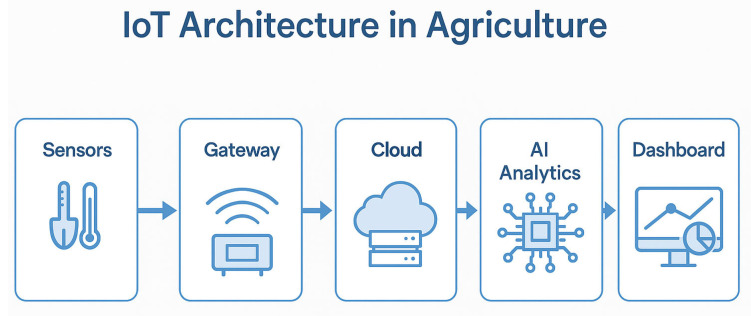
General architecture of an IoT-based agricultural system.

The rapid evolution of IoT-driven agricultural solutions has resulted in the widespread adoption of sensor-based monitoring systems that provide real-time insights into soil conditions, environmental parameters, and plant health [75,76,77]. The effectiveness of AI-driven decision-making models depends heavily on the quality and accuracy of the data obtained from sensor networks deployed across farmlands and grasslands [78,79] (Figure 6)

Optical sensors play a critical role in remote sensing-based crop monitoring. These sensors capture spectral reflectance data [80,81], allowing for vegetation index analysis such as the Normalized Difference Vegetation Index (NDVI) [82], which is commonly used to assess crop health, chlorophyll levels, and drought stress. Satellite-based and UAV-mounted optical sensors are widely used in precision agriculture to detect spatial variations in crop performance and optimize resource distribution [78,83].

Acoustic sensor technology has emerged as a promising tool for detecting pest infestations, soil compaction, and environmental disturbances. These sensors analyze sound wave patterns and vibrations to identify anomalies such as insect activity, animal movement, or soil degradation [84,85]. In precision agriculture, acoustic sensors are particularly useful for monitoring underground soil conditions and assessing the structural integrity of irrigation systems and drainage networks [86,87,88].

Electromagnetic sensors are widely used in precision soil analysis. These sensors measure soil conductivity, salinity, and moisture levels, providing a high-resolution mapping of soil properties across agricultural fields [89,90]. Electromagnetic induction technology enables non-invasive soil characterization, allowing farmers to implement site-specific fertilization and irrigation strategies based on real-time soil variability [91,92,93,94].

Soil and water sensors form the backbone of IoT-enabled smart agriculture, providing critical data on moisture levels, pH balance, temperature, and nutrient content [30,92]. These sensors are crucial for precision irrigation systems, which optimize water usage based on soil moisture availability and real-time weather forecasts. In addition, AI-integrated water sensors help detect contaminants, salinity imbalances, and irrigation inefficiencies, ensuring sustainable water management in agriculture [95,96,97].

Machine learning models play an essential role in analyzing sensor-generated data, enabling farmers to make informed decisions regarding crop health, irrigation schedules, and pest control measures [98,99,100,101]. Supervised learning models, such as random forests and deep neural networks, are commonly used to predict yield outcomes, disease outbreaks, and nutrient deficiencies [102,103,104].

Unsupervised learning approaches, including clustering algorithms and anomaly detection models, help identify patterns and deviations in soil and crop conditions, allowing for early interventions before issues escalate [105,106]. AI-based weather forecasting models further improve precision agriculture strategies, enabling adaptive management plans based on climatic variations [103,107].

The integration of AI-driven decision support systems (DSSs) in agriculture has revolutionized farm management [108,109]. These systems utilize sensor data, remote sensing imagery, and predictive analytics to provide actionable recommendations for irrigation, fertilization, and pest control [83]. IoT-based DSS platforms are designed to be user-friendly and adaptive, allowing farmers to access real-time insights via mobile applications and cloud-based dashboards [110,111].

A comparative overview of sensing technologies used in smart agriculture is presented in Table 2, highlighting key differences in deployment cost, functional effectiveness, durability, and scalability. This classification supports a better understanding of which technologies are suitable for specific applications in precision farming and large-scale arable crop management.

### 3.7. Applications of IoT and AI in Agriculture

Precision irrigation systems utilize real-time soil moisture data, weather predictions, and AI-driven analytics to optimize water distribution across agricultural fields [112,113,114]. IoT-enabled automated drip irrigation and variable-rate irrigation systems ensure that crops receive the exact amount of water required, reducing water wastage and improving crop resilience in drought-prone regions [115,116,117].

AI-enhanced fertilization management relies on soil nutrient analysis, crop growth models, and precision application techniques to ensure optimal nutrient distribution [118,119]. Smart fertilizer dispensers, controlled by IoT sensors, adjust nutrient application based on real-time plant requirements, preventing over-fertilization and minimizing environmental runoff [120,121,122].

AI-powered image recognition and deep learning algorithms enable the real-time detection of pest infestations and plant diseases [123,124,125]. Machine learning models trained on large datasets of crop images can accurately identify symptoms of fungal infections, bacterial diseases, and insect damage [126,127,128]. IoT-connected camera drones and multispectral imaging sensors further enhance disease surveillance by covering large agricultural landscapes efficiently [33,129,130].

IoT-based crop monitoring systems track growth patterns, chlorophyll content, and biomass accumulation through multispectral and hyperspectral imaging technologies [131]. AI-driven growth prediction models help farmers estimate harvest times, assess yield potential, and optimize resource allocation based on growth stage analytics [132,133].

Smart sensing technologies are increasingly being applied to grassland management and urban farming. IoT-powered automated irrigation and climate control systems are being integrated into vertical farming and hydroponic agriculture to optimize urban food production [134]. In rural pasturelands, sensor networks monitor soil quality, grazing patterns, and livestock health [135,136,137] and its rising use in agriculture is reflected in the significant increase in research publications from 2020 onward.

A consolidated overview of key IoT and AI applications in agriculture is provided in Table 3, mapping the type of intelligent model used, sensor deployment strategies, and geographic examples of real-world implementations.

#### Practical Evaluation of Sensing Solutions in Real-World Farming Environments

While sensing technologies demonstrate high potential in experimental and pilot-scale studies, their effectiveness in real-world agricultural environments is influenced by multiple operational constraints. Large-scale commercial farms, particularly in technologically advanced regions, benefit significantly from optical and electromagnetic sensors integrated with UAVs or satellite systems, as these provide high-resolution, spatially continuous data suitable for managing extensive monoculture fields [78,83,92]. However, such systems often require considerable capital investment, stable network infrastructure, and trained personnel for data interpretation, which may be prohibitive for smaller operations [59,60].

In contrast, smallholder farms, especially in resource-constrained regions, tend to adopt simpler, in situ sensing solutions such as soil moisture probes and low-cost IoT water sensors [30,95]. These systems, while limited in spatial coverage, are relatively easy to install and interpret, making them more accessible and appropriate for localized management decisions. Nevertheless, their lower resolution and lack of integration with advanced analytics platforms may restrict their predictive capabilities [138].

Practical limitations also arise from environmental variability. Optical sensors, although highly effective under controlled lighting conditions, are notably sensitive to cloud cover, shadows, and atmospheric interference, which can reduce their accuracy during key crop monitoring periods [80,82,83]. Acoustic sensors, while promising in underground or pest-related detection, may suffer from signal attenuation and noise interference in heterogeneous or waterlogged soil environments [84,85,86]. Moreover, the durability of low-cost sensors remains a concern, as many are not designed to withstand prolonged exposure to extreme weather or mechanical stress [139,140].

These findings underscore the need for context-aware deployment strategies, where sensor selection, network architecture, and AI integration are tailored to the farm size, environmental conditions, and available technical support. Future research and development should focus not only on advancing sensor capabilities but also on improving their robustness, affordability, and adaptability to diverse agricultural settings [141,142,143].

### 3.8. Current Trends and Patterns

The rapid expansion of IoT and AI applications in agriculture has resulted in a sharp increase in peer-reviewed publications between 2022 and 2024, reflecting intensified academic and industrial interest in smart farming solutions. This growth is closely tied to global challenges such as climate change, resource scarcity, and labor shortages, which have pushed the agricultural sector toward technological innovation.

From a geographical perspective, the highest research output is concentrated in countries such as India, China, and Saudi Arabia, driven by large-scale government investments and national digital agriculture strategies. Developed nations, including the United States, Australia, and European countries like Spain and Italy, are also contributing significantly, particularly in domains related to sustainable farming and sensor network deployment [27,55,120].

Technologically, modern agricultural systems increasingly rely on the synergistic use of remote and proximal sensing technologies. Remote sensing, including satellite imagery, UAV-based imaging, and multispectral analysis, provides large-scale data on vegetation patterns, crop health, and land use [144,145,146]. Proximal sensing technologies—such as in situ soil sensors, automated weather stations, and embedded camera networks—offer high-resolution, ground-level data [147,148,149,150]. A key emerging trend is the hybridization of sensing layers, where AI-based data fusion techniques are used to integrate macro- and micro-level inputs for more accurate predictions and real-time interventions [15,151].

In parallel, the agricultural data landscape is undergoing a significant shift toward big data integration and cloud-based AI analytics. Farms now generate terabytes of heterogeneous data, including environmental parameters, sensor readings, and UAV imagery. AI models are increasingly embedded into scalable big data architectures that enable pattern discovery, forecasting, and optimization across entire crop cycles [64,152]. However, the lack of standardized data schemas and communication protocols continues to pose a major barrier to interoperability, prompting initiatives in semantic web technologies and ontology-driven integration frameworks [62,63].

A particularly promising development is the growing implementation of Edge AI—the deployment of machine learning models directly on IoT nodes or edge devices. This approach minimizes latency and reduces reliance on constant cloud connectivity, making it ideal for farms with limited network access [141,153]. Use cases include real-time pest detection using embedded vision modules, on-device irrigation control based on local soil feedback, and event-based anomaly detection in remote fields [154,155]. The use of low-power AI chips and neuromorphic processors is also advancing this area, enabling continuous learning and decision-making, even in energy-constrained environments [156,157].

Unmanned aerial vehicles (UAVs) equipped with AI have also evolved from simple aerial imagers to autonomous diagnostic platforms. Modern drones now carry hyperspectral, multispectral, and thermal cameras, capable of detecting biophysical stressors, nutrient deficiencies, and disease symptoms at high spatial and temporal resolution. With onboard processing power, UAVs can execute deep learning models such as convolutional neural networks (CNNs) or transformer-based classifiers in real time, facilitating precision spraying, targeted scouting, or automated field alerts [27,33,125].

Importantly, this micro-scale, high-frequency analysis is enabling new forms of dynamic and site-specific decision-making. For example, drones can generate actionable maps within a single flight, adjusting irrigation zones or pesticide application areas based on leaf-level indicators. Combined with edge-enabled ground sensors, this forms a closed-loop feedback system, significantly improving the responsiveness and efficiency of agricultural operations.

**Figure 7:**
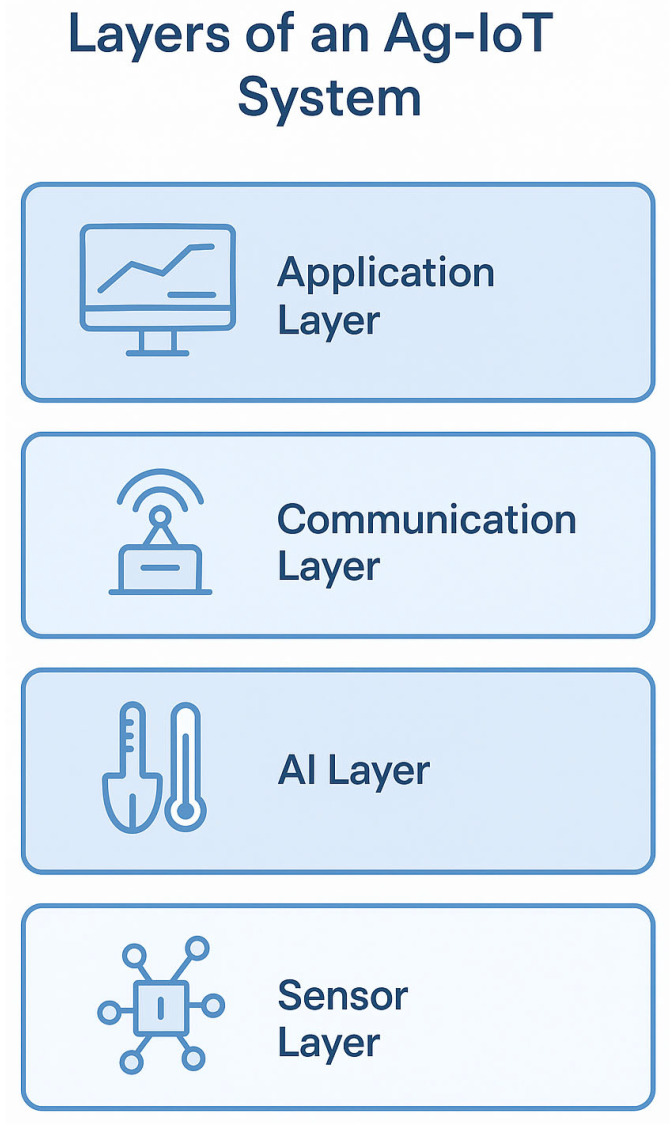
The layered structure of an Ag-IoT system.

These converging trends—AI–big data integration, multi-layered sensing, UAV-based diagnostics, and Edge AI—are redefining the architecture of smart farming. However, for their full potential to be realized, robust regulatory frameworks, sensor interoperability standards, and farmer-centric design principles must be prioritized. The scalability of these technologies will depend not only on their technical merits but also on their affordability, accessibility, and adaptability to diverse agricultural landscapes (Figure 7).

## 4. Problems and Challenges in Smart Agriculture

The integration of the IoT and AI in agriculture has brought significant advancements in crop monitoring, precision irrigation, and automated decision-making [158,159,160]. However, despite the growing adoption of smart sensing technologies, multiple technical, economic, environmental, and ethical challenges continue to hinder their widespread implementation [141,161]. These challenges must be addressed to ensure the long-term viability, accessibility, and effectiveness of AI-driven smart farming solutions.

### 4.1. Technical Barriers

One of the primary technical challenges in smart agriculture is sensor accuracy and calibration variability. Many IoT-based agricultural sensors are designed to measure soil moisture, nutrient levels, temperature, and crop health; however, discrepancies in sensor readings due to environmental conditions, sensor degradation, or manufacturing inconsistencies can lead to unreliable data outputs [162,163]. Inconsistent data significantly affect AI-based decision-making models, as incorrect or noisy inputs can lead to inaccurate predictions, suboptimal irrigation strategies, and inefficient fertilization planning [164,165,166,167].

Moreover, data integration remains a persistent challenge. Agricultural IoT systems generate vast amounts of heterogeneous data from multiple sources, including field sensors, drones, satellite imaging, and weather stations [163,168,169]. Integrating these diverse data streams into a cohesive, real-time decision-support system requires advanced data fusion algorithms and cloud-based computing infrastructures [149,150]. The lack of standardized data formats and interoperability between different IoT devices and AI platforms further complicates seamless data processing and automation in smart agriculture [170,171,172].

Smart agricultural solutions heavily rely on wireless connectivity for real-time data transmission and remote decision-making. However, many rural farming areas, particularly in developing regions, suffer from limited access to broadband internet, weak mobile network coverage, and high latency in data communication [141,173,174]. This lack of connectivity limits the ability of farmers to deploy IoT devices effectively, as real-time monitoring and predictive analytics require continuous data streaming and cloud synchronization [175].

The deployment of low-power wide-area networks (LPWANs), LoRaWAN-based sensors, and satellite internet solutions has helped mitigate some of these issues, but high infrastructure costs and technical expertise requirements remain major barriers to scaling IoT-based precision farming systems in remote regions [176,177,178].

#### AI Model Interpretability and Transparency in Decision-Making

The increasing reliance on AI-driven decision support systems (DSSs) in agriculture introduces the challenge of model interpretability and transparency [179]. While AI algorithms, particularly deep learning and reinforcement learning models, can provide highly accurate predictions for crop yield, disease detection, and irrigation optimization, many of these models function as black-box systems, making it difficult for farmers to understand how decisions are made [180,181,182].

The lack of explainable AI (XAI) frameworks in smart agriculture creates trust issues among farmers, agronomists, and policymakers, who may be reluctant to adopt AI-based recommendations if they cannot verify the rationale behind algorithmic outputs [183,184]. Ensuring model transparency through explainable AI techniques, such as decision trees, SHAP (SHapley Additive Explanations) [185], or LIME (Local Interpretable Model-Agnostic Explanations) [186], is essential to increase the adoption of and trust in AI-based agricultural automation.

### 4.2. Economic and Adoption Barriers

While IoT-based precision agriculture has demonstrated significant benefits in optimizing water usage, fertilizer application, and pest management, the high initial investment cost of deploying smart farming infrastructure remains a major deterrent, especially for smallholder farmers [164,187].

The cost of IoT sensors, automated irrigation systems, AI-based analytics software, and cloud storage services can be prohibitively high, particularly in developing economies where access to financial support is limited. Additionally, regular maintenance, sensor recalibration, and software updates add further operational costs, making IoT adoption financially unfeasible for small farms [136,164,188].

Governments and agricultural organizations have attempted to subsidize smart farming technologies and provide open-source AI models for precision agriculture, but the lack of financial incentives and credit access remains a key factor limiting widespread adoption among resource-constrained farmers [141,165].

Despite the technological potential of the IoT and AI in agriculture, farmer adoption rates remain inconsistent. Many traditional farmers are hesitant to integrate automated decision-making systems into their daily operations due to a lack of digital literacy, fear of technology dependency, and skepticism regarding AI-based recommendations [170,189,190].

Furthermore, agriculture is a deeply traditional industry, and many farmers prefer manual, experience-based approaches over algorithm-driven insights. Limited exposure to AI training programs and user-friendly IoT interfaces has further slowed adoption. Overcoming this resistance requires farmer education programs, hands-on training workshops, and accessible AI interfaces designed for non-technical users [54,191].

### 4.3. Environmental and Sustainability Concerns

The proliferation of IoT devices in agriculture has led to concerns about increased energy consumption and electronic waste. Many smart sensors, wireless networks, and AI computing units require continuous power sources, which can be challenging to sustain in off-grid agricultural regions [141].

Battery-powered IoT devices must be regularly replaced or recharged, leading to higher operational costs and electronic waste accumulation. While solar-powered IoT solutions have been developed to mitigate these concerns, they remain costly and less efficient in regions with inconsistent sunlight availability [30].

Developing low-power AI models, energy-efficient sensor networks, and biodegradable IoT hardware is essential to ensuring the long-term environmental sustainability of smart agriculture [153].

Another sustainability concern involves the long-term viability of sensor networks deployed in agricultural fields. IoT sensors are often exposed to harsh environmental conditions, including extreme temperatures, heavy rainfall, and soil corrosion, which degrade sensor performance over time [139,140].

The frequent replacement of damaged or malfunctioning sensors not only increases operational costs but also contributes to e-waste pollution. Designing rugged, weather-resistant IoT devices with extended lifespans and developing recyclable sensor components are crucial steps in reducing the ecological footprint of precision agriculture technologies [141,192].

### 4.4. Ethical and Policy Challenges

One of the most pressing ethical challenges in AI-driven smart agriculture is data privacy and security. IoT-based farm management systems generate vast amounts of sensitive data, including soil conditions, crop performance, livestock health, and farmer operational strategies. The risk of unauthorized data access, cyberattacks, and corporate exploitation raises concerns about who owns and controls agricultural data [165,180].

Agricultural AI platforms operated by large agritech corporations often require farmers to upload farm data to centralized cloud servers, potentially exposing them to data breaches, unauthorized surveillance, and unfair pricing models dictated by agribusiness monopolies. Implementing secure data encryption, decentralized AI models, and blockchain-based farm record-keeping can enhance data ownership rights and security [193,194].

The lack of standardized regulations governing AI decision-making in agriculture presents a significant policy challenge [171]. Unlike traditional farming practices, where decisions are made by human farmers and agronomists, AI-powered recommendation systems, autonomous irrigation controllers, and robotic harvesters operate with minimal human oversight, raising legal and ethical questions [191].

Governments must establish clear regulatory frameworks that define the accountability, liability, and ethical responsibilities of AI-driven agricultural systems [195]. Regulations should ensure algorithmic transparency, fair AI decision-making, and ethical AI usage, preventing the exploitation of smallholder farmers by proprietary AI-driven agribusiness platforms [139,196].

Despite the transformative potential of the IoT and AI in smart agriculture, several technical, economic, environmental, and ethical barriers must be addressed to ensure fair, transparent, and scalable adoption. The next chapter will explore potential solutions and future research directions, focusing on next-generation AI models, sustainable IoT deployment strategies, and policy interventions for a more inclusive and equitable agricultural future.

To ensure that AI- and IoT-based smart agriculture evolves in an equitable and sustainable manner, policy frameworks must address both technological and socio-economic dimensions. A significant concern is data ownership and access rights, especially when farmers unknowingly generate valuable agronomic data through proprietary platforms. This asymmetry can lead to “data colonialism,” where multinational agritech firms extract and monetize data without fair compensation to local producers.

One promising regulatory approach is the European Union’s Digital Services Act (DSA), which—though not agriculture-specific—provides foundational mechanisms for transparency, accountability, and platform governance. Its principles could be adapted to create agrifood-specific digital legislation, mandating explainable AI, non-discriminatory algorithmic decisions, and data portability for farmers. Importantly, this would enable small- and medium-sized producers to contest decisions made by opaque AI models used in supply chain contracts or subsidy allocations.

In parallel, blockchain technology offers a decentralized alternative to current data handling systems. Immutable ledgers and smart contracts can empower farmers by ensuring that data sharing is consensual, traceable, and securely executed. Pilot projects in India and Kenya have already demonstrated blockchain’s potential for traceability of organic produce, weather-indexed insurance, and peer-to-peer resource trading. However, scalability remains a challenge.

Additionally, policy frameworks must guarantee technology accessibility in the Global South, including public investment in low-cost sensors, open-data platforms, and training programs. Without deliberate policy support, smart agriculture risks deepening the digital divide—benefiting large agribusinesses while excluding smallholder farmers.

Finally, AI audit mechanisms should be established for models used in high-stakes agricultural decision-making (e.g., irrigation control, subsidy qualification). These audits could be modeled on EU AI Act proposals and should include fairness metrics, documentation of training datasets, and bias detection routines.

### 4.5. Cybersecurity Risks in AI- and IoT-Enabled Agriculture

As agricultural systems become increasingly digitized, cybersecurity emerges as a critical concern. Most IoT-based sensor networks currently used in agriculture lack basic encryption protocols, leaving data streams vulnerable to interception, manipulation, or spoofing. Many low-cost sensors and communication modules transmit unencrypted data packets, especially over wireless sensor networks (WSNs), making them susceptible to eavesdropping and tampering by malicious actors [141,171].

Furthermore, the decentralized and heterogeneous nature of agricultural IoT infrastructures—often composed of devices from multiple vendors—creates vulnerabilities that can be exploited through backdoors, firmware weaknesses, or unsecured communication protocols. Remote attackers may target gateways, edge nodes, or cloud synchronization points to inject false sensor readings, disable irrigation control systems, or trigger unnecessary pesticide dispersal [162,197].

In large-scale farm deployments, such breaches can result in significant economic damage, data loss, or unintended environmental harm. For example, spoofed temperature or moisture readings could lead to incorrect AI-based decisions, affecting crop yield or resource efficiency. This highlights the urgent need for built-in end-to-end encryption, regular firmware updates, and secure device authentication mechanisms.

Beyond hardware-level risks, data-level threats also pose serious challenges. AI models used for decision-making are only as reliable as the data they ingest. If adversarial inputs or corrupted sensor streams are fed into machine learning pipelines, they can distort predictive outcomes and lead to flawed recommendations. In this context, anomaly detection algorithms play a vital role—not only in identifying natural outliers but also in detecting potential cyber-induced data anomalies [180,185].

Future research should focus on the integration of AI-based intrusion detection systems (IDSs) tailored for agriculture, capable of flagging suspicious activity in real time. These systems must be lightweight, compatible with edge infrastructure, and designed with the specific constraints of agricultural environments in mind.

## 5. Future Perspectives and Recommendations

The rapid advancement of the IoT and AI technologies in agriculture has revolutionized how farmers monitor crops, optimize irrigation, and mitigate risks related to climate variability and pest infestations [171,198]. However, the successful transition to fully automated and AI-driven smart farming systems depends on overcoming technical, economic, and policy-related challenges [199,200]. The future of the IoT and AI in agriculture lies in developing next-generation smart sensing technologies, integrating decentralized AI models, enhancing sustainability, and creating clear regulatory frameworks to ensure ethical AI implementation [201,202].

### 5.1. Next-Generation IoT and AI Technologies in Agriculture

The next wave of IoT and AI-driven agricultural solutions is expected to focus on increasing sensor precision, reducing energy consumption, and enabling real-time data analytics with minimal infrastructure requirements [203]. The future of precision farming will likely integrate high-resolution hyperspectral imaging, low-power AI chips, and self-learning AI models that continuously improve based on farm-specific conditions [141,142].

One of the most promising advancements is the development of AI-enhanced multi-sensor fusion systems. These systems will combine optical, acoustic, electromagnetic, and soil sensors into integrated monitoring platforms capable of providing highly detailed, real-time insights into soil health, moisture levels, and plant physiology [143,204,205]. Instead of relying on individual sensors with isolated functionalities, next-generation multi-modal IoT networks will use AI-driven data fusion to extract deeper insights and optimize agricultural decision-making [92,95,203].

Additionally, advancements in neuromorphic computing [156,206] and AI-on-a-chip solutions will enable low-power AI models that can process data locally without requiring constant cloud connectivity [153,157]. These embedded AI processors will support real-time on-field decision-making, making smart farming more resilient to connectivity disruptions and reducing the dependence on centralized cloud services [207].

### 5.2. Emerging Trends: Edge AI, Blockchain, and Robotics

Several emerging technologies are poised to redefine smart agriculture, including Edge AI, blockchain-based farm data management, and autonomous robotic farming systems. These innovations will enhance efficiency, transparency, and sustainability in IoT-enabled agricultural ecosystems [197,208,209].

One of the major limitations of traditional AI-based smart farming systems is their reliance on cloud computing, which requires continuous internet connectivity and high-bandwidth data transfers. Edge AI, which processes data locally on the farm instead of sending them to remote servers, offers a more efficient and scalable alternative [154,162].

Edge AI-powered IoT sensors and drones can analyze real-time crop images, detect pest infestations, and optimize irrigation schedules without requiring external data processing units. This low-latency decision-making capability is particularly beneficial for rural farms with poor internet connectivity. As AI hardware becomes more power-efficient and cost-effective, Edge AI will become a cornerstone of autonomous precision agriculture [155,210].

Blockchain technology has the potential to revolutionize agricultural data management by ensuring secure, tamper-proof, and decentralized record-keeping [208]. One of the biggest challenges in AI-driven agriculture is the centralization of farm data, where large agribusiness corporations control sensor-generated insights, predictive analytics, and farm operational data. Farmers often lack ownership over their own data, leaving them vulnerable to exploitation, price manipulation, and the loss of autonomy [211,212].

By integrating blockchain-powered smart contracts, farmers can retain full control over their data, ensuring that AI algorithms operate transparently and that predictive models are not biased toward proprietary agribusiness interests [208]. Additionally, blockchain-enabled traceability can help verify organic certification, food safety compliance, and fair-trade standards, ensuring that farmers receive fair market value for their products [200].

The use of AI-powered robotic systems in agriculture is expanding rapidly, with automated drones, self-driving tractors, and robotic harvesters becoming increasingly sophisticated [1,213]. AI-powered agricultural robots can perform precision weeding, autonomous planting, and selective harvesting, reducing reliance on manual labor and chemical herbicides [214].

The future of agricultural robotics will focus on developing AI-enhanced robotic swarms, where multiple autonomous machines work collaboratively to manage large farms [215,216]. These robotic networks will use reinforcement learning algorithms to optimize crop maintenance, soil health monitoring, and yield predictions, making fully autonomous farms a reality in the coming decades [217].

### 5.3. Bridging the Gap Between Research and Implementation

Despite the technological advancements in smart agriculture, a significant gap remains between academic research and real-world adoption. Many AI-powered agricultural solutions are developed in research laboratories and controlled test environments, but their scalability and adaptability to diverse farming conditions are often overlooked [218,219,220].

One of the key challenges is the lack of collaboration between AI researchers, agronomists, and farmers [221]. Many AI models are designed without sufficient input from agricultural experts, leading to misalignment between theoretical AI predictions and real-world farming constraints. Establishing interdisciplinary collaborations between data scientists, agronomists, and policymakers is crucial to ensuring that AI-driven smart farming solutions are practical, scalable, and tailored to farmers’ needs [222].

Additionally, many smallholder farmers struggle with limited access to digital infrastructure, funding, and technical expertise, preventing them from adopting AI-driven agricultural technologies [223]. Bridging this gap requires government-backed financial incentives, farmer education programs, and accessible open-source AI platforms that can be customized to different agricultural environments [218,219].

Another critical aspect is standardization and interoperability. The lack of universal IoT communication protocols and standardized data-sharing frameworks prevents farmers from integrating multiple smart farming solutions into a cohesive system [62,152,224]. Developing open-source agricultural AI models and IoT interoperability standards will ensure that farmers can adopt smart farming solutions without being locked into proprietary ecosystems controlled by a few major agritech corporations [225,226].

### 5.4. Policy and Ethical Considerations in Smart Farming

The widespread adoption of AI and the IoT in agriculture raises several ethical and regulatory concerns, particularly related to data privacy, algorithmic fairness, and corporate monopolization. Policymakers must establish clear legal frameworks to protect farmers’ rights, ensure ethical AI deployment, and prevent AI-driven exploitation in the agricultural sector [221,227].

As AI-powered decision-making systems become more prevalent in agriculture, ensuring algorithmic transparency is crucial. AI models used for yield prediction, irrigation management, and pest detection must be explainable, auditable, and free from biased decision-making [180,223]. Regulatory bodies must enforce explainability standards, requiring agritech companies to provide interpretable AI outputs rather than black-box predictions [228].

Data privacy is another major concern. Many IoT-based farm management platforms require farmers to upload sensitive farm data to centralized cloud services, leaving them vulnerable to corporate surveillance, unauthorized data exploitation, and unfair pricing strategies [227,229]. Governments must implement strong data protection regulations that give farmers full control over their agricultural data, ensuring that AI-driven insights are used ethically and equitably [180,230].

The rise in AI-powered agribusiness platforms has the potential to reshape global food supply chains, but it also raises the risk of market monopolization and price manipulation [83]. Large corporations could use AI-driven market predictions to dictate commodity prices, favor large-scale industrial farms, and marginalize smallholder farmers. Regulatory policies must ensure fair AI-driven agricultural markets, preventing the use of AI for anti-competitive practices that disadvantage local farmers [20,231,232].

The future of the IoT and AI in agriculture depends on technological innovation, interdisciplinary collaboration, and ethical policy development. Advancements in Edge AI, blockchain, and autonomous robotics will drive the next generation of smart farming solutions, but addressing adoption barriers, regulatory challenges, and farmer education gaps is essential to achieving sustainable, equitable, and transparent AI-driven agriculture. The next steps must focus on scaling AI solutions, making smart farming affordable, and ensuring that digital agriculture benefits all farmers—regardless of farm size or economic status.

## 6. Conclusions

The integration of the IoT and AI has revolutionized modern agriculture by enabling real-time monitoring, predictive analytics, and automation across key farming operations. Smart sensing systems and machine learning models now support precision irrigation, early disease detection, and optimized fertilization. Despite these technological gains, several persistent barriers remain, including technical limitations, economic inaccessibility, environmental costs, and ethical concerns.

Our systematic review highlights a rapid increase in high-quality research on AI and IoT in agriculture between 2020 and 2024, particularly in technologically advanced countries. Yet, the uneven global distribution of innovation reflects a digital divide, with smallholder and resource-constrained farmers having limited access to smart solutions. Connectivity issues, the high cost of sensor infrastructure, and the lack of interoperable platforms further hinder inclusive adoption.

From a technological standpoint, developments in Edge AI, low-power sensors, and blockchain for secure data handling show strong potential to increase scalability and resilience. However, several critical research gaps remain. Notably, there is a lack of field-based validation for AI-driven sensing systems in complex environments such as agroforestry or intercropping systems—particularly in tropical and subtropical regions. Additionally, the absence of open-source, vendor-neutral frameworks limits data interoperability, benchmarking, and cross-regional deployment, especially in low- and middle-income countries.

Ethical and cybersecurity considerations—such as data privacy, model transparency, and system resilience—are still under-addressed in many deployments. The rise in decentralized architectures, including Edge AI and autonomous UAV systems, makes it urgent to embed anomaly detection, encryption, and explainable AI methods directly into smart agricultural systems.

To support a sustainable and inclusive digital farming future, future efforts must prioritize the following:

Energy-efficient and biodegradable sensing systems;

Transparent AI governance policies (e.g., informed by frameworks like the European Digital Services Act);

Affordable open platforms enabling smallholder participation.

In conclusion, the transformative potential of AI and the IoT in agriculture can only be fully realized through interdisciplinary collaboration, ethical implementation, and equitable access. Addressing current gaps will be essential to building resilient, scalable, and sustainable food systems for the decades to come.

## Figures and Tables

**Table 1:** PRISMA flow summary table.

Screening Stage	Number of Records	Notes
Records identified through database search	585	Scopus, Web of Science, IEEE Xplore, SpringerLink, Google Scholar
Records removed (non-English)	–10	Language exclusion
Records removed (not open access)	–415	Open-access filter applied
Records remaining after filtering	160	Only OA papers considered
Records removed (non-peer-reviewed, gray lit.)	–48	Only journal articles and conference papers retained
Studies included in the final review	112	Used in analysis and discussion

**Table 2:** Comparison of sensing technologies in smart agriculture.

Technology Type	Typical Applications	Deployment Cost	Effectiveness	Durability	Scalability
Optical Sensors	Remote crop monitoring, NDVI	Medium to High	High for above-ground vegetation indices	Moderate (weather-sensitive)	High (e.g., satellite/UAV-based)
Acoustic Sensors	Pest detection, soil compaction, drainage	Low to Medium	Moderate—depends on signal clarity	High (few moving parts)	Moderate (requires calibration)
Electromagnetic Sensors	Soil conductivity, moisture mapping	Medium	High precision in subsurface soil data	High (non-invasive)	Moderate (field-based setup)
Soil/Water Sensors	Irrigation control, nutrient balance	Low to Medium	High—real-time, in situ data	Variable (sensor-specific)	High (commonly deployed in WSNs)

**Table 3:** Applications of IoT and AI in smart agriculture.

Application Area	AI Model/Algorithm	Sensor Type	Case Study/Region
Precision Irrigation	Random Forest, SVM	Soil moisture sensors, WSNs	India [55], USA [51]
Fertilizer Optimization	KNN, Decision Trees	Soil nutrient sensors, UAV imagery	Saudi Arabia [120], Spain [118]
Pest and Disease Detection	Convolutional Neural Networks	Multispectral imaging, camera UAVs	China [125], Colombia [21]
Crop Monitoring and Yield Estimation	Deep Learning, LSTM	Optical sensors, NDVI, drones	Australia [27], Italy [131]
Livestock Grazing/Grassland Monitoring	Clustering (K-means), Rule-based	Soil probes, GPS collars, drones	Ireland [35], Kenya [135]
Urban Smart Grasslands	Image Recognition + IoT Fusion	Fixed camera networks, soil sensors	Germany [38], Japan [137]

## Data Availability

No new data were created during the preparation of the manuscript.

## Abbreviations

The following abbreviations are used in this manuscript:

AI	Artificial Intelligence
IoT	Internet of Things
ML	Machine Learning
DL	Deep Learning
WSN	Wireless Sensor Network
NDVI	Normalized Difference Vegetation Index
DSS	Decision Support System
XAI	Explainable Artificial Intelligence
LPWAN	Low-Power Wide-Area Network
LoRaWAN	Long Range Wide Area Network
UAV	Unmanned Aerial Vehicle
SHAP	SHapley Additive Explanations
LIME	Local Interpretable Model-Agnostic Explanations
Edge AI	AI Processing at the Edge (On-Device Computing)
AIoT	Artificial Intelligence of Things (AI + IoT)
ICT	Information and Communication Technology
GPS	Global Positioning System
RFID	Radio-Frequency Identification
IoE	Internet of Everything
GIS	Geographic Information System
PRISMA	Preferred Reporting Items for Systematic Reviews and Meta-Analyses
